# Editorial: Variable autonomy for human-robot teaming

**DOI:** 10.3389/frobt.2024.1465183

**Published:** 2024-11-06

**Authors:** Andreas Theodorou, Manolis Chiou, Bruno Lacerda, Simon Rothfuß

**Affiliations:** ^1^ Department of Computer Science, Universitat Politecnica de Catalunya, Barcelona, Spain; ^2^ School of Electronic Engineering and Computer Science, Queen Mary University of London, London, United Kingdom; ^3^ Oxford Robotics Institute, University of Oxford, Oxford, United Kingdom; ^4^ Institute of Control Systems, Karlsruhe Institute of Technology, Karlsruhe, Germany

**Keywords:** variable autonomy, shared control, human-robot teaming, human-robot interaction, mixed-initiative, human-initiative, human-machine cooperation, shared autonomy

## Introduction

In the modern era, the integration of robots has become a cornerstone of progress in various sectors, from entertainment and companionship to first responders and defence. One of the most critical aspects of this integration is the concept of Human-Robot Teaming (HRT). Unlike traditional automation, where robots operate in isolation or perform predefined tasks, HRT involves robots working alongside humans and aiming to achieve shared goals, often in dynamic and interactive environments. This collaboration leverages the strengths of both humans and robots to achieve goals that neither could accomplish as effectively alone.

Such capabilities often involve teaming with well-defined and static control frameworks: humans *in-*, *on-*, or *out-of-the-*loop. However, as the world is not static and more complex tasks are being required of HRT, the ability to dynamically allocate tasks with varying levels of autonomy becomes essential. Variable Autonomy (VA) refers to the ability of the robotic systems to dynamically vary their level or degree of autonomy to collaborate with the human(s) efficiently and based on the context of overcoming various challenging circumstances.

This Research Topic, dedicated to Variable Autonomy, encompasses such research, including but not limited to shared control and shared autonomy, mixed-initiative, adjustable autonomy, sliding autonomy, and adaptive automation. This topic is driven by the need to bring together VA-related research and practices often disconnected across different communities, as the field is relatively young.

The four original research articles in this Research Topic speak to two major challenges relating to variable autonomy research:

1. How to perform efficient and trustworthy task allocation in HRT.
2. How users perceive robots in HRT and perform autonomy adjustment.

## Tasks allocation

Human-robot teams must handle a diverse array of tasks of variable complexity, from gross and fine motor skills to visual perception, cognitive processing, and speech. Such tasks may coincide or occur in quick succession. For efficient teaming, robots must be able to identify these composite, concurrent tasks performed by humans.

Baskaran and Adams review over a hundred task recognition algorithms and evaluate them on six criteria: sensitivity, suitability, generalizability, composite factor, concurrency, and anomaly awareness. Through the extensive review (Baskaran and Adams), make multiple recommendations for future directions, including the need for ecologically valid HRT datasets and adaptively segmenting metrics.

The need for more efficient metrics in HRT is tackled by another paper in our Research Topic (Verhagen et al.). propose an evaluation method to verify if dynamic task allocation using variable autonomy in human-robot teams ensures not just completion of the task but also *meaningful human control* by satisfying accountability, responsibility, and transparency. This approach quantifies traceability both subjectively and objectively by analysing human responses during and after simulated collaborative activities. Additionally, it incorporates semi-structured interviews following the simulation to uncover the underlying reasons for the outcomes and gathers suggestions for enhancing the variable autonomy strategy. In their article, a real-world illustration with firefighters is presented.

## Robot perception and autonomy adjustment

Lakhnati et al. examine the use of Large Language Models (LLMs) to facilitate variable autonomy through verbal human-robot communication. They present a virtual reality simulator for HRT with variable autonomy studies and simulated robot agents with an LLM as their controller for autonomy switching. Their works indicate that the use of an LLM as a sole controller leads to multiple limitations primarily due to their non-deterministic nature and latency issues from cloud connectivity. Their user study suggests that users often see these robots as less than equal conversational partners, leading to interactions limited to basic commands. However, they also observed that users without preconceived notions about the robots’ conversational limitations engaged in more complex dialogues.

In “*Event-Triggered Robot Self-Assessment to Aid in Autonomy Adjustment*” (Conlon et al.), propose a framework for robotic in-mission confidence self-assessment, which can be reported to a human operator who then uses the information to determine the appropriate level of autonomy for the human-robot team. The framework is based on two key components: the Model Quality Assessment (MQA), a metric that assesses how well the robot’s model aligns with reality; and an Event-Triggered algorithm which monitors the MQA and triggers a more thorough Generalized Outcome Assessment of the robot’s confidence in achieving high-level task objectives.

## Conclusion

In this Research Topic, the published studies contribute to the understanding of Variable Autonomy for Human-Robot Teaming in a timely manner. Although Variable Autonomy spans many disciplines with a variety of challenges remaining unresolved, the papers in this Research Topic focus mainly on two aspects: 1) How to perform efficient and trustworthy task allocation in HRT via Variable Autonomy and 2) How users perceive robots and perform autonomy adjustments in HRTs. We hope this Research Topic will be a starting point for consolidation in the Variable Autonomy community.

